# A Phase I clinical trial of carbon ion radiotherapy for Stage I breast cancer: clinical and pathological evaluation

**DOI:** 10.1093/jrr/rry113

**Published:** 2019-02-26

**Authors:** Kumiko Karasawa, Tokuhiki Omatsu, Atsushi Arakawa, Naohito Yamamoto, Takashi Ishikawa, Mitsue Saito, Shigekazu Fukuda, Tadashi Kamada, Working Group for Breast Cancer

**Affiliations:** 1 National Institute of Radiological Sciences, National Institutes for Quantum and Radiological Science and Technology, 4-9-1, Anagawa, Inage-ku, Chiba-city, Japan; 2 Department of Radiation Oncology, Tokyo Women’s Medical University, 8-1 Kawada-cho, Shinjuku-ku, Tokyo, Japan; 3 Department of Pathology, Juntendo University, 2-1-1, Hongo, Bunkyo-ku, Tokyo, Japan; 4 Department of Breast Surgery, Chiba Cancer Center,666-2, Nitonacho, Chuo-ku, Chiba-city, Japan; 5 Department of Breast Surgery, Tokyo Medical University, 6-1-1, Shinjuku, Shinjuku-ku, Tokyo, Japan; 6 Department of Breast Surgery, Juntendo University, 2-1-1, Hongo, Bunkyo-ku, Tokyo, Japan

**Keywords:** carbon ion radiotherapy, breast cancer, Phase I clinical trial, hypofractionated radiotherapy, pathological evaluation

## Abstract

Even with its high RBE and >20 years history, there had been no breast cancer clinical trial using carbon-ion radiotherapy. We started a Phase I trial of carbon ion radiotherapy for Stage I breast cancer in 2013. This article describes the clinical and pathological evaluation of this study. Patients with low-risk Stage I breast cancer were eligible. A dose escalation study was designed, with dose levels of 48.0, 52.8 or 60.0 Gy relative biological effectiveness (RBE) administered in four fractions within 1 week. Three months after radiotherapy, the patients underwent tumor excision for pathological evaluation. Between April 2013 and December 2014, three cases receiving 48 Gy (RBE), three cases receiving 52.8 Gy (RBE) and one case receiving 60 Gy (RBE) underwent this protocol. No adverse effects were observed except for Grade 1 acute skin reaction in four cases. Pathological evaluation revealed that all four cases with doses of 52.8 Gy (RBE) and 60.0 Gy (RBE) achieved Grade 2b or more, but only two cases reached Grade 3. At the end of 2017, all cases were alive without recurrence or late had not caused any late adverse reaction. Carbon ion radiotherapy for Stage I breast cancer seems to be safe, and we found that it did not reach enough treatment effect 3 months after the treatment.

## INTRODUCTION

Carbon ion radiotherapy is known to have a high relative biological effectiveness (RBE) and a target-concentrated dose distribution. The National Institute of Radiological Sciences (NIRS) in Japan commenced the clinical use of carbon ion radiotherapy in 1994, and has treated more than 10 000 tumors in the last 24 years and conducted >80 clinical trials for a variety of malignant tumors [1]. The clinical trials have had two types of focus: (i) the treatment for conventionally radioresistant tumors, such as bone and soft tissue sarcomas and malignant melanomas; and (ii) low-burden treatment of common cancers, such as 1-day treatment for Stage I lung cancer and 2-day treatment for hepatocellular carcinoma.

Breast cancer is the most common cancer in women, but as at 2013 it had not been taken up as a protocol study of carbon ion radiotherapy. The reason is that earlier researchers had not identified the types of breast cancer suitable for localized irradiation; in addition, they thought that the standard treatment for breast cancer was not a heavy burden for patients. However, accelerated partial-breast irradiation came into practice for low-risk tumors recently, and the development of minimally invasive therapy is particularly important in an aging society. In addition, some patients want treatment for breast cancer without surgery. Some clinical trials of non-surgical therapies, such as radiofrequency ablation (RFA) [2–4], cryoablation therapy [5–7], and high-intensity focused ultrasound (HIFU) have been conducted [8–10]. The clinical data available for these therapies are still not sufficient with respect to adaptations, optimal procedures, verification of methods, number of cases, clinical trials, and follow-up periods.

The NIRS had treated only four breast cancer patients with carbon ion radiotherapy prior to 2013: three cases of chemoresistant metastatic lung tumor and one case of metastatic liver tumor were treated. To our best knowledge, no institute has reported a clinical trial of carbon ion radiotherapy for breast cancer.

Due to carbon ion radiotherapy’s high RBE and high linear energy transfer (LET), it has been shown that the dose fractionation effect is small. Commencing with Stage I lung cancers, we started to treat lung tumors with 72.0 Gy (RBE) in nine fractions in 1999, then gradually reduced the fraction number to four fraction, with a dose of 52.8 Gy (RBE) from 2003 [11, 12]. The overall local control rate obtained was 96.3%, with acceptable toxicities.

Based on the efficacy and safety of carbon ion radiotherapy for various kinds of malignancies, it is meaningful to conduct a clinical trial of carbon ion radiotherapy for primary breast tumor, not with post-operative intent, but with radical intent. In 2011, the Breast Cancer Study Group of the NIRS commenced preparation for the clinical trial. The group consisted of radiation oncologists, radiologists, breast surgical oncologists, and pathologists who specialized in breast cancer pathology. In 2013, we started a Phase I/II clinical trial of hypofractionated carbon ion radiotherapy for low-grade Stage I breast cancer with radical intent. This article reports the clinical and pathological results of the Phase I clinical trial.

## MATERIALS AND METHODS

The purpose of this Phase I clinical trial for low-risk Stage I breast cancer was to conduct targeted treatment with carbon ion radiotherapy in four fractions administered over 1 week, to confirm its safety and efficacy and to clarify the recommended dose for the Phase II trial. In Phase I, it was planned that patients underwent primary tumor excision and sentinel lymph node biopsy for pathological evaluation 3 months after the carbon ion radiotherapy. This protocol was approved by the ethics committee of the NIRS (ID 1301) and registered with the University hospital Medical Information Network (UMIN ID 000010848) and the Japan Breast Cancer Society (Clinical Trial No.77).

### Eligibility criteria

Eligibility criteria for the patients were as follows: (i) having pathologically proven invasive ductal carcinoma of the breast; (ii) having a solitary tumor with a diameter ≤ 2 cm as revealed in MRI, including ductal spread, UICC Stage I (T1N0M0); (iii) having no extensive lymphatic invasion and no extensive intraductal component, and being estrogen receptor (ER) positive and HER2 negative; (iv) having a performance status of 0–2; (v) being a woman of ≥60 years of age; (vi) having a life expectancy of >6 months; (vii) being willing to participate in the clinical trial and having provided written informed consent. In addition, ineligibility criteria were as follows: (i) having severe complications that could prevent the treatment being tolerated (e.g. uncontrolled cardiopulmonary disease, intractable infectious disease, uncontrolled mental illness); (ii) having a history of treatment of the present breast cancer; (iii) being under systemic drug therapy for active double cancer; (iv) having a tumor with chest wall or skin invasion; (v) having a distance between the tumor and the skin, including the intraductal component, able to be identified on the MRI as <5 mm; (vi) having a history of radiotherapy to the irradiation site; (vii) having pathology of non-invasive ductal carcinoma (pure DCIS); (viii) the attending physician considered participation inappropriate due to psychological or other factors. Every candidate was considered carefully by the Breast Tumor Protocol Operational Board regarding eligibility. Radiation strategies for all approved patients were deliberated on and approved at the Carbon Ion Radiotherapy Conference.

### Carbon ion radiotherapy

**Fig. 1.**
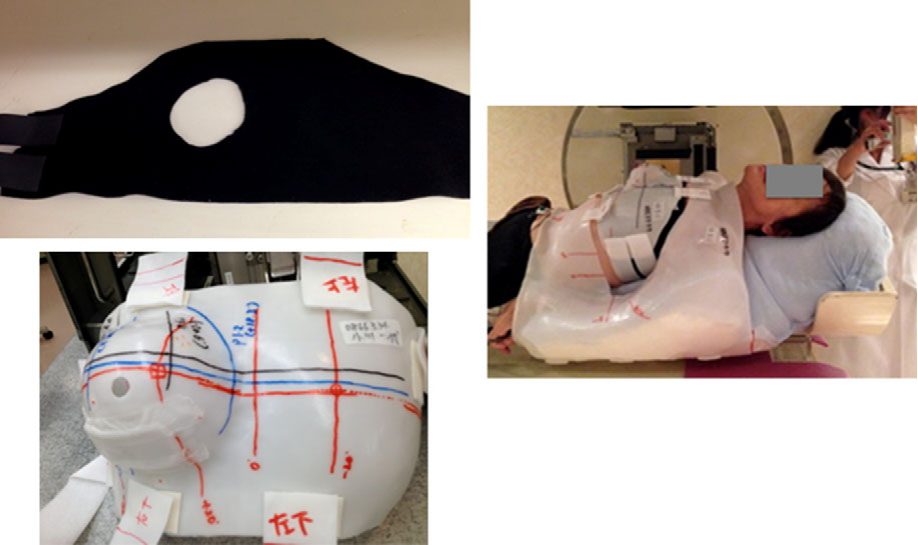
Immobilization devices and treatment positioning.

Carbon ion radiotherapy was performed using the heavy-ion medical accelerator in Chiba (HIMAC) at NIRS. Each patient was immobilized using a cast (Mold care; Alcare, Tokyo, Japan), a breast belt made of elastic fibers (in order to compress the contralateral breast and to project the affected breast), and a thermoplastic body fixture shell (Shellfitter; Kerary Co., Ltd, Osaka, Japan)(Fig. 1). A prone breast position was not used because of the physical limitations of the treatment couch. Before taking the treatment planning images, two fiducial markers (Visicoil^TM^; IBA, Schwarzenbruck, Germany) were inserted for position recognition at 5 mm from the upper border and 5 mm from the lower border of the ductal spread under ultrasound (US) guidance. Two Visicoils of 5 mm long and 0.5 mm diameter were used, and the dose attenuation impact of the carbon ion beam had been measured before treatment (using phantoms) and confirmed as being within the neglect dose range.

**Fig. 2.**
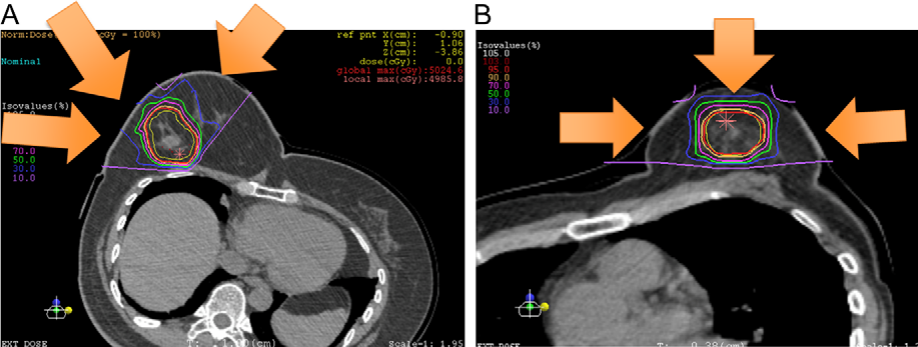
Portal setting and dose distribution of the patients: (A) No. 3 case; (B) No. 5 case.

The details of the treatment planning have been reported previously [13]. The gross tumor volume (GTV) was defined as the volume of the tumor based on contrast MRI findings. The clinical target volume (CTV) was defined as the area containing the GTV plus the intraducral components of the tumor. The planning target volume (PTV) was defined as the region CTV plus a safety margin of 5 mm ( to allow for any inaccuracy due to geometric variations). This ensured that the dose to the skin did not exceed 30Gy(RBE) per week and was <50% of the prescription dose. Irradiation was carried out using a 290 MeV carbon ion beam from three ports to the front–left–right of the target (Fig. 2). The irradiation dose was decided (based on the results of clinical trials of carbon ion radiotherapy) as 4 times the irradiation dose administered for Stage I lung cancer. The dose escalation of this trial was carried out at the following three dose levels: Level 1: 12.0 Gy (RBE) four times a week, total 48.0 Gy (RBE); Level 2: 13.2 Gy (RBE) four times a week, total 52.8 Gy (RBE), Level 3: 15.0 Gy (RBE) four times a week, total 60.0 Gy (RBE).

### End points

The primary end point was an acute adverse effect, and the secondary end points were tumor control, a late adverse effect, cosmetic outcome, disease-free survival and overall survival. Acute adverse effects occurring within 90 days from carbon ion radiotherapy were observed and recorded using NCI Common Terminology Criteria for Adverse Events, Version 4.0 (CTC-AE v4) [14] at the end of treatment, 2 weeks, 1 month, 2 months and 3 months after treatment. The treatment observation period was concluded at up to 3 months after carbon ion radiotherapy, and the follow-up period was concluded at up to 5 years after carbon ion radiotherapy. Dose escalation could be carried out without dose-limiting toxicity (DLT) for 6 months after the carbon ion radiotherapy in three cases. The DLT was defined as radiation dermatitis of Grade 3 or higher; respiratory symptoms (such as pneumonitis) of Grade 3 or higher, nausea; vomiting and fatigue of Grade 4; and performance status 4. In Phase I, no additional treatment was performed during the 3 months following the conclusion of the carbon ion radiotherapy. Treatment effect judgments were performed based on the MRI and US images at 1 month and 3 months after the carbon ion radiotherapy, and the pathological effect on the resected tumor was determined at 3 months after the carbon ion radiotherapy. After tumor resection, endocrine therapy was initiated as a standard adjuvant treatment. The follow-up studies performed included physical exanimation, US and MRI at least every 6 months. The initial biopsy specimens were stained with hematoxylin and eosin (HE), and the resected tumor specimens were examined by the pathologist in our group. The pathological treatment effects were judged in accordance with Oboshi–Shimosato classification (Grade 0: cancer cell damages are not noted, Grade 1: but cancer nets have been destroyed, Grade 2: cancer cell damage, cancer nets have been bestroyed, and Grade 3: non-viable cancer cells are present) [15]. Late effects were assessed based on the RTOG/EORTC Late Radiation Morbidity Scoring System [16]. Cosmetic outcome was checked every month during the observation period and once every 6 months during the follow-up period with reference to breast size, shape, scar, hardness, the position of the nipple compared with the untreated breast, and graded as poor/fair/good/excellent. The primary end point was an acute adverse effect, and the secondary end points were local control rate, late reaction, cosmetic outcome, and overall survival.

## RESULTS

A total of seven patients were registered in this study from July 2013 to December 2014, including three cases of Level 1 dose, three cases of Level 2 dose and one case of Level 3 dose (Table 1). After registration of the seventh patient, there were no further registrations after 1 year and 3 months, so we decided to finalize the protocol in April 2016.

**Table 1.** Cases in Phase I clinical trial

Case No.	Age	Tumor size (mm)	Dose, Gy (RBE)	Skin reaction	3-month effect (MRI/US)	3-month pathological effect^a^	Endocrine therapy	Follow-up (months)	Prognosis
1	66	12	48	1	PR	G1a	Refusal	40	NED
2	63	9	48	0	SD	G0	AI	48	NED
3	61	20	48	1	CR	G3	AI	46	NED
4	76	14	52.8	0	PR	G2b	TAM	43	NED
5	61	4	52.8	1	PR	G3	TAM	43	NED
6	66	13	52.8	1	PR	G2b	AI	40	NED
7	81	20	60.0	0	PR	G2b	AI	37	NED

^a^Oboshi–Shimozato classification. PR = pathological response, SD = stable disease, CR = complete response, AI = aromatase inhibitor, TAM = tamoxifen, NED = no evidence of disease.

**Fig. 3.**
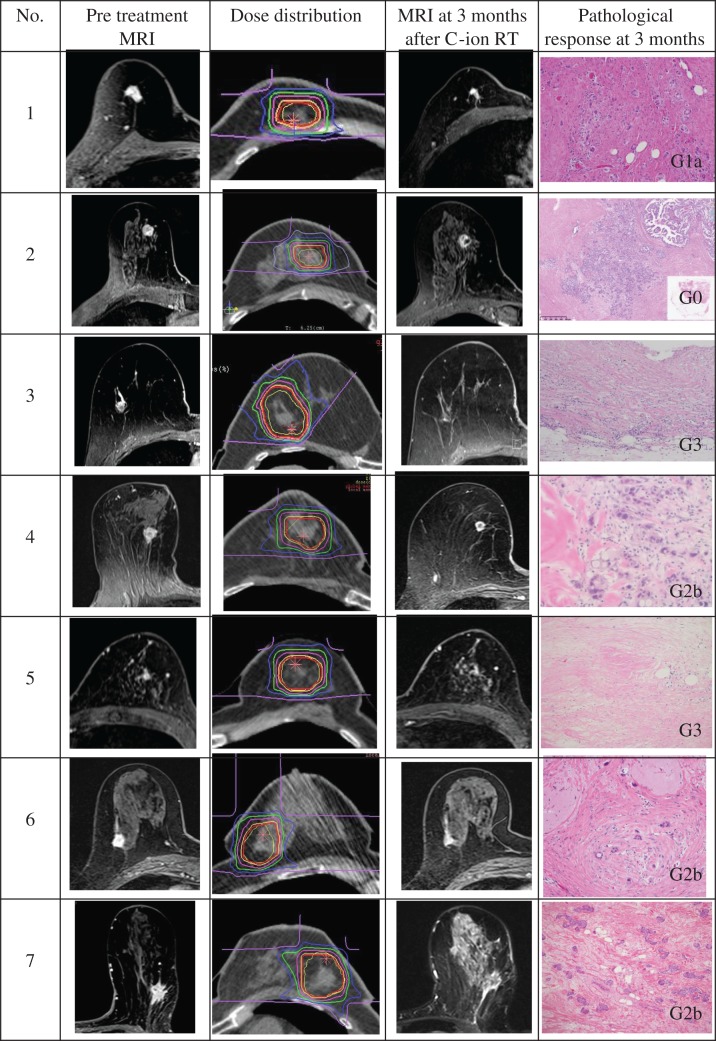
Pre-treatment tumor on contrast-enhanced gradient-echo T1-weighted MRI, the dose distribution of C-ion at the central part of the tumor, MRI at 3 months after carbon ion radiotherapy and HE-stained tissue specimen and pathological treatment effect valuation at 3 months after carbon ion radiotherapy.

The age of the patients ranged from 61 to 81, and the tumor size ranged from 4 mm to 20 mm. All patients were ER positive, progesterone receptor (PgR) positive and HER2 negative. No adverse reactions were observed except for a Grade 1 acute skin reaction in four cases lasting a few weeks. Evaluation based on MRI and US at 1 month after the carbon ion radiotherapy indicated two patients with partial response (PR) and five patients with stable disease (SD); at 3 months after carbon ion radiotherapy, one patient was found to have complete response (CR), five PRs and one SD. All tumors were resected with negative surgical margins. Based on pathological evaluation, one patient’s tumor was assessed as Grade 0, one as Grade 1a, three as Grade 2b and two as Grade 3. At the level 1 dose: 48 Gy (RBE) of carbon ion radiotherapy, one patient’s tumor was assessed as Grade 0, another patient’s as Grade 1a, and the third patient’s as Grade 3. At the level 2 dose: 52.8 Gy (RBE), two cases were assessed as Grade 2b, and 1 case was assessed as Grade 3. The patient’s tumor irradiated with 60.0 Gy (RBE) was assessed as Grade 2b. The tumor image on contrast-enhanced gradient-echo T1-weighted MRI at pre-treatment and 3 months after carbon ion radiotherapy, the dose distribution resulting from the carbon ion irradiation at the central part of the tumor, and an HE-stained tissue specimen 3 months after carbon ion radiotherapy are shown in Fig. 3. Endocrine therapy was conducted in six cases, but one patient refused to take this medication. Four cases were administered aromatase inhibitor, whereas the other two cases were administered tamoxifen due to osteoporosis. At the end of 2017 (a follow-up period of 37–48 months), all cases were alive, with excellent cosmetic outcome, without recurrence and without any late effects.

## DISCUSSION

Several institutes have been reporting on the application of particle therapy for breast cancer; however, those reports concern proton therapy as part of postoperative adjuvant therapy [17–20]. To the best of our knowledge, the only articles on carbon ion radiotherapy for breast cancer in the literature are two case reports from NIRS [13, 21].

Based on NIRS’s clinical experiences, the therapeutic effect of carbon ion radiotherapy on breast cancer was expected to be similar to that for lung cancer, so we planned tumor resection for pathological evaluation 3 months after the carbon ion radiotherapy. A 3-month period was expected to be adequate for pathological evaluation; however, at 3 months the tumor cells were still alive in 5 of 7 cases. We wondered whether this meant that carbon ion radiotherapy was not as effective for breast cancer as it was for various other malignancies. The patients with Stage I breast cancer that we treated in parallel with our Phase I clinical trials indicated that this was not the case. The NIRS have a 24-year history of using carbon ion radiotherapy, and from the outset we have treated various malignancies using a ‘general protocol’. We employed the general protocol known as ‘advanced medical care’ (AMC) for patients who were inoperable for physical and/or psychological reasons and who could thus not fulfil the clinical trial criteria.

When we announced the commencement of the Phase I trial, many patients with Stage I breast cancer applied; however, most of them did not fulfil the application criteria due to age, pathological subtype or unsuitability for surgery. If they had the reason that they could not fit in standard treatment, we offered them the AMC protocol program on ethical grounds. AMC patients did not have to undergo tumor resection, unlike the patients in the clinical trials, but they had to pay for treatment costs.

The case of the first AMC patient was reported by Akamatsu *et al.* [13]. It took >1 year for the tumor to disappear on the MRI/US image. The details of 14 stage I AMC cases will be reported in another paper, but here we note that it took >6 months to show the effect of the carbon ion radiotherapy. On this basis, we decided that the clinical significance of pathological evaluation after 3 months was low and we discontinued the Phase I trial after the seventh case, and decided to start the Phase II trial at the 60.0 Gy (RBE) dose level.

Although this report concerns only seven cases over a 3-month period only, there have been few reports to date of pathological examination post carbon ion radiotherapy, so we believe this report will be valuable. From the results of this study, the dose of 48 Gy (RBE) over four fractions was deemed to be relatively ineffective as a treatment dose, even though one case reached Grade 3, one reached Grade 1 and one reached Grade 0 over the 3-month period. For the doses of 52.8 Gy (RBE) and 60.0 Gy (RBE), all cases reached Grade 2b or better, and 60.0 Gy (RBE) was determined to be the recommended dose due to the very mild acute adverse effects. Initially, we considered dose escalation to 66 Gy (RBE) after observing the results of the Phase I trial. However, we observed that the therapeutic effect of 60 Gy (RBE) at AMC was good, and thus considered it unnecessary to increase the dose to 66 Gy (RBE).

There are other options for non-surgical treatment for early-stage breast cancer, but they are usually accompanied by pain that requires the addition of general anesthesia to the procedure, and the tumor is slow to disappear. Carbon ion radiotherapy is less invasive: the only invasive procedure is insertion of a marker under local anesthesia. Since the tumor softens just 1 month after treatment, and cannot be palpated after only ~3 months, the psychological burden on the patient is small; adverse events are also minor.

The problems with using carbon ion radiotherapy are the cost and the technical difficulties involved in the construction and maintenance of treatment facilities. However, these problems will be solved by researchers of physics engineering. Already, facilities about one-third of the size and less than half the cost of HIMAC facilities have been made and put into operation [22]. Scanning irradiation that can be adapted to accommodate respiratory movement and which uses a rotating gantry is now being practised at NIRS.

In this article, we report the results of the first Phase I trial of carbon ion radiotherapy for breast cancer. Carbon ion radiotherapy for patients with Stage I breast cancer promises to be useful and not to have problematic adverse effects, although it might take a longer period to achieve a complete response for breast cancer than for lung cancer. We believe that clinical research on carbon ion radiotherapy for breast cancer should be continued in preparation for the day when this treatment will be accessible to many patients.
